# 4-Phenyl-1,2,4-tri­aza­spiro­[4.5]dec-1-ene-3-thione

**DOI:** 10.1107/S1600536813019120

**Published:** 2013-07-13

**Authors:** Mehmet Akkurt, Joel T. Mague, Shaaban K. Mohamed, Alaa A. Hassan, Mustafa R. Albayati

**Affiliations:** a Department of Physics, Faculty of Sciences, Erciyes University, 38039 Kayseri, Turkey; b Department of Chemistry, Tulane University, New Orleans, LA 70118, USA; c Chemistry and Environmental Division, Manchester Metropolitan University, Manchester M1 5GD, England; d Chemistry Department, Faculty of Science, Mini University, 61519 El-Minia, Egypt; e Kirkuk University, College of Science, Department of Chemistry, Kirkuk, Iraq

## Abstract

In the title compound, C_13_H_15_N_3_S, the 4,5-di­hydro-3*H*-1,2,4-triazole ring is nearly planar [maximum deviation = 0.020 (1) Å], while the cyclo­hexane ring adopts a chair conformation. The dihedral angle between the 4,5-di­hydro-3*H*-1,2,4-triazole ring and the phenyl ring is 74.68 (7)°. No specific inter­molecular inter­actions are discerned in the crystal packing.

## Related literature

For wide-spectrum medicinal applications of spiro compounds incorporating heterocyclic substructures, see: Patil *et al.* (2010 ▶); Pawar *et al.* (2009 ▶); Thadhaney *et al.* (2010 ▶); Chin *et al.* (2008 ▶); Wang *et al.* (2007 ▶); Chande *et al.* (2005 ▶); Obniska *et al.* (2006 ▶); Kamiński *et al.* (2008 ▶); Sarma *et al.* (2010 ▶); Shimakawa *et al.* (2003 ▶). For ring-puckering parameters, see: Cremer & Pople (1975 ▶).
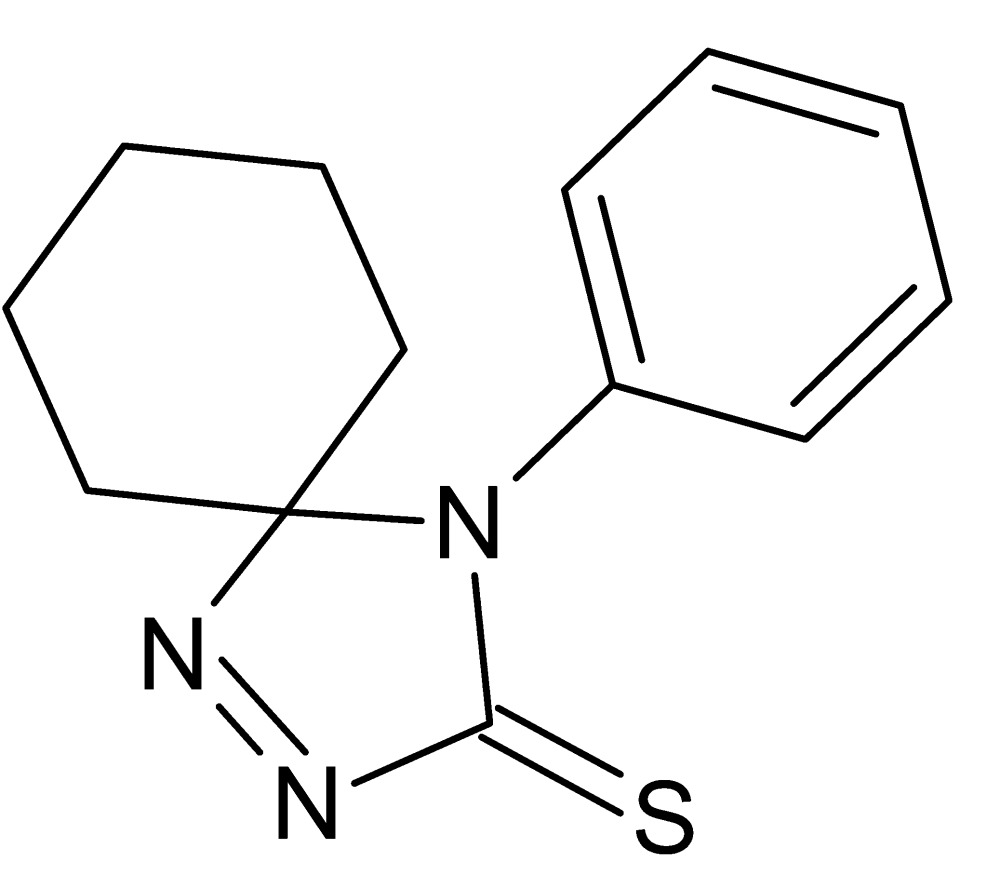


## Experimental

### 

#### Crystal data

C_13_H_15_N_3_S
*M* *_r_* = 245.34
Orthorhombic, 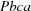

*a* = 9.4952 (9) Å
*b* = 7.4845 (7) Å
*c* = 34.692 (3) Å
*V* = 2465.5 (4) Å^3^
*Z* = 8
Mo *K*α radiation
μ = 0.24 mm^−1^
*T* = 150 K
0.28 × 0.22 × 0.17 mm

#### Data collection

Bruker SMART APEX CCD diffractometer
Absorption correction: multi-scan (*SADABS*; Bruker, 2013 ▶) *T*_min_ = 0.810, *T*_max_ = 0.960
41202 measured reflections
3168 independent reflections
2899 reflections with *I* > 2σ(*I*)
*R*_int_ = 0.046

#### Refinement

*R*[*F* ^2^ > 2σ(*F* ^2^)] = 0.042
*wR*(*F* ^2^) = 0.104
*S* = 1.10
3168 reflections
154 parameters
H-atom parameters constrained
Δρ_max_ = 0.40 e Å^−3^
Δρ_min_ = −0.25 e Å^−3^

### 

Data collection: *APEX2* (Bruker, 2013 ▶); cell refinement: *SAINT* (Bruker, 2013 ▶); data reduction: *SAINT*; program(s) used to solve structure: *SHELXS97* (Sheldrick, 2008 ▶); program(s) used to refine structure: *SHELXL97* (Sheldrick, 2008 ▶); molecular graphics: *PLATON* (Spek, 2009 ▶); software used to prepare material for publication: *publCIF* (Westrip, 2010 ▶).

## Supplementary Material

Crystal structure: contains datablock(s) global, I. DOI: 10.1107/S1600536813019120/tk5237sup1.cif

Structure factors: contains datablock(s) I. DOI: 10.1107/S1600536813019120/tk5237Isup2.hkl

Supplementary material file. DOI: 10.1107/S1600536813019120/tk5237Isup3.cml

Additional supplementary materials:  crystallographic information; 3D view; checkCIF report

## Figures and Tables

**Fig. 1.**
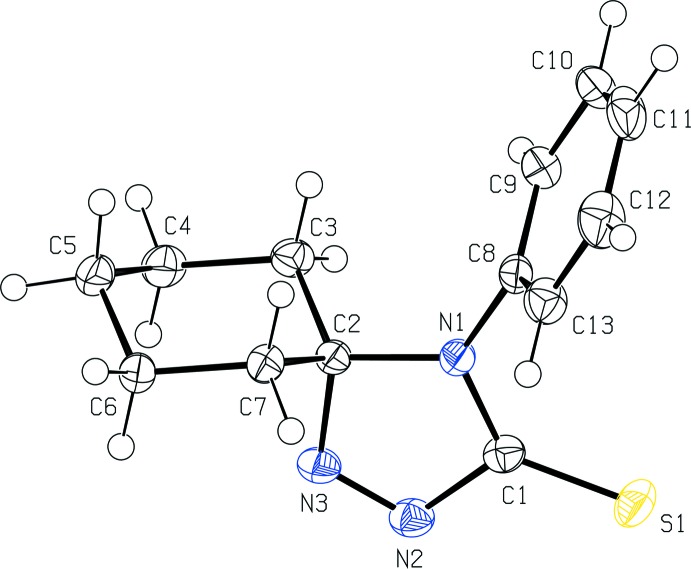
View of the title compound (I) with the atom numbering scheme. Displacement ellipsoids for non-H atoms are drawn at the 50% probability level.

## supplementary crystallographic information

### Comment

Heterocyclic compounds such as 1.2.4-triazoles exhibit a wide range of biological activities (Patil *et al.*, 2010). Several spiro-compounds incorporating different heterocyclic structures have showed significant industrial and pharmaceutical applications such as anti-microbial (Pawar *et al*., 2009; Thadhaney *et al.*, 2010), anti-cancer (Chin *et al*., 2008; Wang *et al.*, 2007), anti-tubercular (Chande *et al*., 2005) and anti-convulsant activities (Obniska *et al.*, 2006; Kamiński *et al.*, 2008) as well as functioning as antioxidants (Sarma *et al.*, 2010; Shimakawa *et al.*, 2003). In view of such facts and as part of our on-going study on the synthesis of bio-active molecules, we herein report the synthesis and crystal structure of the title compound (I).

As shown in Fig. 1, the 4,5-dihydro-3*H*-1,2,4-triazole ring (N1–N3/C1/C2) of (I) is nearly planar with a maximum deviation of 0.020 (1) Å for N1 and it makes a dihedral angle of 74.68 (7)° with the phenyl ring (C8–C13). The cyclohexane ring (C2–C7) adopts a chair conformation with the puckering parameters (Cremer & Pople, 1975) of Q_T_ = 0.5610 (14) Å, θ = 1.74 (14)° and φ = 342 (4)°.

The stabilization of the molecular packing of (I) is assisted by a number of non-bonded forces including van der Waals.

### Experimental

A solution of 2-cyclohexylidene-*N*-phenylhydrazinecarbothioamide (1 mmol) in dry ethyl acetate (15 ml) was added drop wise over 2 h to a stirred solution of 4,5-dichloro-3,6-dioxocyclohexa-1,4-diene-1,2-dicarbonitrile (227 mg, 1 mmol) in dry ethyl acetate (10 ml). The pink coloration of the reaction mixture turned quickly to red brown and the mixture was left to stand at room temperature for 48 h. The precipitated DDQ-H_2_ [JM1] was filtered off and washed with few drops of ethyl acetate. The filtrate was collected, concentrated under vacuum and left at room temperature to afford the title compound as red brown crystals suitable for X-ray diffraction.

IR: 3052 (Ar—CH), 2941, 2863 (Ali – CH), 1594 (Ar—C=C), 1350 (C═S); ^1^H-NMR (CDCl_3_) 1.26, 1.65, 1.94 and 2.20 (10*H*, cyclohexane–CH_2_), 7.707, 7.51, 7.55 (5H, Ar–H); ^13^C–NMR (CDCl_3_) 23.58, 24.52 and 33.90 (cyclohexane-CH_2_), 112.19 (spiro Cx b), 127.81, 129.87, 130.22 (Ar—CH), 135.07 (Ar-c), 187.83 (C═S).

### Refinement

All H atoms were placed in geometrically idealized positions and constrained to ride on their parent atoms with C—H = 0.95 and 0.99 Å, with *U*_iso_(H) = 1.2 *U*_iso_(C).

### Crystal data

**Table:**

C_13_H_15_N_3_S	*F*(000) = 1040
*M**_r_* = 245.34	*D*_x_ = 1.322 Mg m^−^^3^
Orthorhombic, *P**b**c**a*	Mo *K*α radiation, λ = 0.71073 Å
Hall symbol: -P 2ac 2ab	Cell parameters from 9973 reflections
*a* = 9.4952 (9) Å	θ = 2.5–28.6°
*b* = 7.4845 (7) Å	µ = 0.24 mm^−^^1^
*c* = 34.692 (3) Å	*T* = 150 K
*V* = 2465.5 (4) Å^3^	Block, red-brown
*Z* = 8	0.28 × 0.22 × 0.17 mm

### Data collection

**Table:**

Bruker SMART APEX CCD diffractometer	3168 independent reflections
Radiation source: fine-focus sealed tube	2899 reflections with *I* > 2σ(*I*)
Graphite monochromator	*R*_int_ = 0.046
Detector resolution: 8.3660 pixels mm^-1^	θ_max_ = 28.7°, θ_min_ = 2.4°
φ and ω scans	*h* = −12→12
Absorption correction: multi-scan (*SADABS*; Bruker, 2013)	*k* = −10→10
*T*_min_ = 0.810, *T*_max_ = 0.960	*l* = −46→46
41202 measured reflections	

### Refinement

**Table:**

Refinement on *F*^2^	Primary atom site location: structure-invariant direct methods
Least-squares matrix: full	Secondary atom site location: difference Fourier map
*R*[*F*^2^ > 2σ(*F*^2^)] = 0.042	Hydrogen site location: inferred from neighbouring sites
*wR*(*F*^2^) = 0.104	H-atom parameters constrained
*S* = 1.10	W = 1/[Σ^2^(*F*O^2^) + (0.0483*P*)^2^ + 1.0447*P*] where *P* = (*F*O^2^ + 2*F*C^2^)/3
3168 reflections	(Δ/σ)_max_ = 0.001
154 parameters	Δρ_max_ = 0.40 e Å^−^^3^
0 restraints	Δρ_min_ = −0.25 e Å^−^^3^

### Special details

**Table:**

Geometry. Bond distances, angles *etc*. have been calculated using the rounded fractional coordinates. All su's are estimated from the variances of the (full) variance-covariance matrix. The cell e.s.d.'s are taken into account in the estimation of distances, angles and torsion angles
Refinement. Refinement on *F*^2^ for ALL reflections except those flagged by the user for potential systematic errors. Weighted *R*-factors *wR* and all goodnesses of fit *S* are based on *F*^2^, conventional *R*-factors *R* are based on *F*, with *F* set to zero for negative *F*^2^. The observed criterion of *F*^2^ > σ(*F*^2^) is used only for calculating -*R*-factor-obs *etc*. and is not relevant to the choice of reflections for refinement. *R*-factors based on *F*^2^ are statistically about twice as large as those based on *F*, and *R*-factors based on ALL data will be even larger.

### Fractional atomic coordinates and isotropic or equivalent isotropic displacement parameters (Å^2^)

**Table:**

	*x*	*y*	*z*	*U*_iso_*/*U*_eq_	
S1	0.53002 (4)	1.40579 (5)	0.84564 (2)	0.0310 (1)	
N1	0.50442 (11)	1.06645 (14)	0.87249 (3)	0.0199 (3)	
N2	0.53364 (11)	1.27385 (15)	0.91860 (3)	0.0252 (3)	
N3	0.51796 (11)	1.13018 (15)	0.93661 (3)	0.0233 (3)	
C1	0.52216 (12)	1.24250 (17)	0.87698 (4)	0.0219 (3)	
C2	0.49469 (12)	0.97820 (16)	0.91027 (3)	0.0188 (3)	
C3	0.60868 (13)	0.83732 (18)	0.91727 (4)	0.0241 (3)	
C4	0.59589 (15)	0.75735 (19)	0.95773 (4)	0.0282 (4)	
C5	0.44880 (15)	0.68348 (18)	0.96539 (4)	0.0280 (4)	
C6	0.33484 (14)	0.82248 (17)	0.95781 (3)	0.0240 (3)	
C7	0.34706 (13)	0.90101 (16)	0.91725 (3)	0.0209 (3)	
C8	0.47802 (13)	0.97938 (17)	0.83642 (3)	0.0208 (3)	
C9	0.58094 (15)	0.87142 (18)	0.82019 (4)	0.0273 (4)	
C10	0.55240 (17)	0.7858 (2)	0.78557 (4)	0.0346 (4)	
C11	0.42447 (19)	0.8099 (2)	0.76734 (4)	0.0377 (5)	
C12	0.32316 (18)	0.9190 (2)	0.78367 (4)	0.0369 (4)	
C13	0.34905 (15)	1.00429 (19)	0.81847 (4)	0.0283 (4)	
H3A	0.70270	0.89270	0.91420	0.0290*	
H3B	0.59970	0.74110	0.89790	0.0290*	
H4A	0.66580	0.66010	0.96070	0.0340*	
H4B	0.61770	0.85060	0.97710	0.0340*	
H5A	0.43240	0.57840	0.94860	0.0340*	
H5B	0.44270	0.64330	0.99250	0.0340*	
H6A	0.34290	0.91980	0.97700	0.0290*	
H6B	0.24110	0.76650	0.96090	0.0290*	
H7A	0.27600	0.99650	0.91390	0.0250*	
H7B	0.32760	0.80660	0.89800	0.0250*	
H9	0.66940	0.85640	0.83260	0.0330*	
H10	0.62130	0.71000	0.77430	0.0410*	
H11	0.40610	0.75150	0.74360	0.0450*	
H12	0.23540	0.93560	0.77100	0.0440*	
H13	0.27940	1.07870	0.82980	0.0340*	

### Atomic displacement parameters (Å^2^)

**Table:**

	*U*^11^	*U*^22^	*U*^33^	*U*^12^	*U*^13^	*U*^23^
S1	0.0373 (2)	0.0214 (2)	0.0344 (2)	−0.0050 (1)	0.0002 (1)	0.0067 (1)
N1	0.0234 (5)	0.0179 (5)	0.0183 (5)	−0.0008 (4)	−0.0003 (4)	0.0003 (4)
N2	0.0254 (5)	0.0214 (5)	0.0287 (6)	−0.0028 (4)	0.0010 (4)	−0.0045 (4)
N3	0.0255 (5)	0.0209 (5)	0.0235 (5)	−0.0009 (4)	−0.0004 (4)	−0.0056 (4)
C1	0.0190 (5)	0.0195 (6)	0.0272 (6)	−0.0014 (4)	0.0011 (4)	−0.0011 (5)
C2	0.0230 (5)	0.0172 (6)	0.0162 (5)	−0.0001 (4)	−0.0014 (4)	−0.0013 (4)
C3	0.0240 (6)	0.0248 (6)	0.0235 (6)	0.0054 (5)	−0.0030 (5)	−0.0015 (5)
C4	0.0337 (7)	0.0276 (7)	0.0234 (6)	0.0075 (6)	−0.0077 (5)	0.0000 (5)
C5	0.0406 (7)	0.0218 (6)	0.0215 (6)	0.0034 (5)	−0.0025 (5)	0.0022 (5)
C6	0.0308 (6)	0.0208 (6)	0.0204 (6)	−0.0017 (5)	0.0018 (5)	0.0011 (4)
C7	0.0216 (5)	0.0200 (5)	0.0210 (6)	−0.0004 (5)	−0.0011 (4)	0.0011 (4)
C8	0.0263 (6)	0.0189 (6)	0.0172 (5)	−0.0034 (5)	0.0008 (4)	0.0014 (4)
C9	0.0313 (7)	0.0269 (6)	0.0236 (6)	0.0007 (5)	0.0058 (5)	0.0003 (5)
C10	0.0504 (9)	0.0281 (7)	0.0252 (7)	−0.0031 (6)	0.0146 (6)	−0.0030 (5)
C11	0.0607 (10)	0.0340 (8)	0.0185 (6)	−0.0168 (7)	0.0013 (6)	−0.0025 (5)
C12	0.0434 (8)	0.0404 (8)	0.0268 (7)	−0.0093 (7)	−0.0107 (6)	0.0008 (6)
C13	0.0302 (7)	0.0298 (7)	0.0250 (6)	−0.0013 (5)	−0.0029 (5)	0.0006 (5)

### Geometric parameters (Å, º)

**Table:**

S1—C1	1.6375 (14)	C11—C12	1.383 (2)
N1—C1	1.3375 (17)	C12—C13	1.388 (2)
N1—C2	1.4706 (15)	C3—H3A	0.9900
N1—C8	1.4330 (15)	C3—H3B	0.9900
N2—N3	1.2525 (16)	C4—H4A	0.9900
N2—C1	1.4669 (17)	C4—H4B	0.9900
N3—C2	1.4757 (16)	C5—H5A	0.9900
C2—C3	1.5305 (17)	C5—H5B	0.9900
C2—C7	1.5354 (17)	C6—H6A	0.9900
C3—C4	1.531 (2)	C6—H6B	0.9900
C4—C5	1.525 (2)	C7—H7A	0.9900
C5—C6	1.5239 (19)	C7—H7B	0.9900
C6—C7	1.5293 (15)	C9—H9	0.9500
C8—C9	1.3874 (19)	C10—H10	0.9500
C8—C13	1.3864 (19)	C11—H11	0.9500
C9—C10	1.388 (2)	C12—H12	0.9500
C10—C11	1.381 (2)	C13—H13	0.9500
			
C1—N1—C2	110.28 (10)	H3A—C3—H3B	108.00
C1—N1—C8	124.87 (11)	C3—C4—H4A	109.00
C2—N1—C8	124.27 (10)	C3—C4—H4B	109.00
N3—N2—C1	110.17 (10)	C5—C4—H4A	109.00
N2—N3—C2	111.76 (10)	C5—C4—H4B	109.00
S1—C1—N1	131.58 (11)	H4A—C4—H4B	108.00
S1—C1—N2	122.06 (10)	C4—C5—H5A	109.00
N1—C1—N2	106.36 (11)	C4—C5—H5B	109.00
N1—C2—N3	101.32 (9)	C6—C5—H5A	109.00
N1—C2—C3	113.98 (10)	C6—C5—H5B	109.00
N1—C2—C7	111.53 (9)	H5A—C5—H5B	108.00
N3—C2—C3	109.08 (9)	C5—C6—H6A	109.00
N3—C2—C7	109.21 (9)	C5—C6—H6B	109.00
C3—C2—C7	111.19 (10)	C7—C6—H6A	109.00
C2—C3—C4	111.03 (10)	C7—C6—H6B	109.00
C3—C4—C5	111.97 (11)	H6A—C6—H6B	108.00
C4—C5—C6	111.88 (11)	C2—C7—H7A	109.00
C5—C6—C7	111.55 (10)	C2—C7—H7B	109.00
C2—C7—C6	111.04 (10)	C6—C7—H7A	110.00
N1—C8—C9	119.74 (11)	C6—C7—H7B	109.00
N1—C8—C13	119.05 (11)	H7A—C7—H7B	108.00
C9—C8—C13	121.21 (11)	C8—C9—H9	120.00
C8—C9—C10	118.85 (13)	C10—C9—H9	121.00
C9—C10—C11	120.50 (14)	C9—C10—H10	120.00
C10—C11—C12	120.08 (13)	C11—C10—H10	120.00
C11—C12—C13	120.31 (15)	C10—C11—H11	120.00
C8—C13—C12	119.04 (13)	C12—C11—H11	120.00
C2—C3—H3A	109.00	C11—C12—H12	120.00
C2—C3—H3B	109.00	C13—C12—H12	120.00
C4—C3—H3A	110.00	C8—C13—H13	121.00
C4—C3—H3B	109.00	C12—C13—H13	120.00
			
C2—N1—C1—S1	−175.88 (10)	N2—N3—C2—C7	−116.28 (11)
C2—N1—C1—N2	3.52 (12)	N1—C2—C3—C4	177.48 (10)
C8—N1—C1—S1	−4.33 (19)	N3—C2—C3—C4	65.07 (13)
C8—N1—C1—N2	175.07 (10)	C7—C2—C3—C4	−55.43 (13)
C1—N1—C2—N3	−3.14 (12)	N1—C2—C7—C6	−175.60 (10)
C1—N1—C2—C3	−120.14 (11)	N3—C2—C7—C6	−64.44 (12)
C1—N1—C2—C7	112.94 (11)	C3—C2—C7—C6	55.98 (13)
C8—N1—C2—N3	−174.76 (10)	C2—C3—C4—C5	54.36 (15)
C8—N1—C2—C3	68.24 (14)	C3—C4—C5—C6	−53.80 (15)
C8—N1—C2—C7	−58.67 (14)	C4—C5—C6—C7	54.12 (14)
C1—N1—C8—C9	110.43 (14)	C5—C6—C7—C2	−55.15 (13)
C1—N1—C8—C13	−69.89 (16)	N1—C8—C9—C10	178.89 (12)
C2—N1—C8—C9	−79.16 (15)	C13—C8—C9—C10	−0.8 (2)
C2—N1—C8—C13	100.51 (14)	N1—C8—C13—C12	−179.57 (12)
C1—N2—N3—C2	0.53 (13)	C9—C8—C13—C12	0.1 (2)
N3—N2—C1—S1	176.89 (9)	C8—C9—C10—C11	1.0 (2)
N3—N2—C1—N1	−2.58 (13)	C9—C10—C11—C12	−0.5 (2)
N2—N3—C2—N1	1.50 (12)	C10—C11—C12—C13	−0.2 (2)
N2—N3—C2—C3	122.02 (11)	C11—C12—C13—C8	0.4 (2)
